# Effect of Small Molecular Additives on Growth Rates of Molecular Crystals from the Melt near Glass-Transition Temperature

**DOI:** 10.1021/acs.cgd.5c01407

**Published:** 2025-12-15

**Authors:** Alexander G. Shtukenberg, Hengyu Zhou, Eli Finkelstein, Aminata Dioume

**Affiliations:** Department of Chemistry and Molecular Design Institute, 5894 New York University, New York, New York 10003, United States

## Abstract

Frequently, small concentrations of additives can substantially modify crystal growth rates from solutions, while a substantial concentration of additives is required to likewise affect crystallization from the melt. Recently, it was discovered that even 1 wt % of polymeric additive can significantly either increase or decrease growth rates of molecular crystals depending on the differences between glass-transition temperatures *T*_g_ of host crystals and polymers [C. Huang et al., *J. Phys. Chem. B* **2017**, 121, 1963–1971]. A similar effect is illustrated here for small-molecule additives. It is shown that the growth rate of a molecular crystal increases if the *T*_g_ of an additive is lower than that of a host crystal and growth rate decreases if the *T*_g_ of an additive is higher than that of a host crystal. This effect is observed only for host crystals that can form intermolecular hydrogen bonds in the melt. We speculate that in this case, the additive can efficiently disrupt the hydrogen bond network and affect the liquid dynamics in the melt.

## Graphical abstract

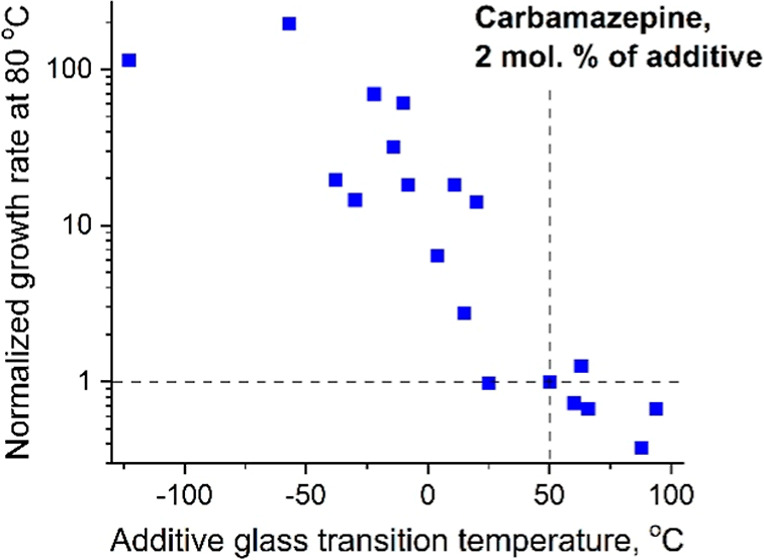

## Introduction

Ability to control crystallization is important for various areas of science and industry. Some examples include the formulation of amorphous drugs^1–4^ or specific polymorphs,^5^ prevention of pathological mineralization in humans^6,7^ and animals,^8^ increasing the lethality of contact insecticides^9–11^ as well as the prevention of ice crystallization in food^12^ and tissues.^13^ Crystallization is performed in different media, mostly from solutions and from the melt/amorphous solid. Here, we focus only on crystallization from the melt.

Crystallization can often be controlled by chemical additives. So-called tailor-made additives, however, are not very efficient in melt crystallization.^14^ Instead, amorphous solid dispersions are commonly used in the pharmaceutical industry to stabilize amorphous drugs. They typically contain about 5–20 wt % of active pharmaceutical ingredient (API) dispersed in a polymer.^1–4^ In this case, the API molecules are separated by polymer chains and strong inhibition of crystal growth and nucleation is easy to rationalize. Surprisingly, addition of only 1 wt % of a polymer can substantially decrease or increase the linear growth rate of small molecular crystals.^15–17^ The direction and magnitude of the growth rate change strongly depend on the difference between the glass-transition temperature, *T*_g_, of the host crystal and the polymer additive. Such growth rate changes were connected to the relation between a polymer segmental mobility and a host dynamic, but no further analysis has been performed.^16^

Inspired by this research, we asked whether a small fraction of small molecular additives can induce substantial change in the growth rate of a molecular crystal from the melt. We focused on small molecules for a few reasons. First, it is experimentally simpler to probe the effect of a variety of functional groups on the growth rate. Second, understanding polymers is more difficult since it has to include analysis of the backbone dynamics, and we wanted to eliminate these complications. Finally, controlling crystallization with small molecules provides additional opportunities for potential practical applications. We discovered that near the host *T*_g_ some small molecule hosts exhibit dramatic growth rate changes up to 4 orders of magnitude at an additive concentration of 2 mol %, while other hosts remain unaffected. We speculate such a phenomenon results from the additive disrupting the structure of a supercooled liquid, forming regions with higher or lower molecular mobility.

## Experimental Section

Carbamazepine 98%, sulfapyridine 99%, paracetamol 98%, coumarin 99%, antipyrine 97%, benzamide 99%, nicotinamide 99.5%, isonicotinamide 99%, tolbutamide 97%, flufenamic acid 97%, tolfenamic acid 98%, (+)-griseofulvin 97%, theophylline 99%, chlorpropamide 97%, deltamethrin (analytical standard), α-cypermethrin (analytical standard), sulfathiazole (no purity provided), dimethyl sulfoxide (DMSO, 99.9%), and ethanol (200 proof) were purchased from Sigma-Aldrich. 5-Methyl-2-[(2-nitrophenyl) amino]-3-thiophenecarbonitrile (ROY, 97%) was purchased from TCI America, chlorfenapyr 98% was purchased from Santa Cruz Biotechnology, and salicylic acid (no purity provided) was purchased from Mallinckrodt. All chemicals were used without any purification. Some properties of molecules studied are shown in Table 1; their molecular structures are shown in Figure S1.

**Table 1:** Properties (Molecular Weight *M*_w_, Density ρ, Melting Point *T*_m_, and Glass-Transition Temperature *T*_g_) of the Materials Studied^d^

compound	molecular formula	*M*_w_, g/mol	ρ, g/cm^3^	*T*_m_, °C^a^	*T*_g_, °C	ref^b^	H-donor^c^
*hosts*							
chlorfenapyr	C_15_H_11_BrClF_3_N_2_O	407.62	1.69	101	–15	^18^	No
ROY	C_12_H_9_N_3_O_2_S	259.29	1.43	94	–14	^19^	No
α-cypermethrin	C_22_H_19_Cl_2_NO_3_	416.30	1.37	79	–9	*	No
deltamethrin	C_22_H_19_Br_2_NO_3_	505.20	1.52	100	–9	*	No
paracetamol	C_8_H_9_NO_2_	151.16	1.30	169	25	^20^	Yes
carbamazepine	C_15_H_12_N_2_O	236.27	1.34	191	50	^21^	Yes
sulfapyridine	C_11_H_11_N_3_O_2_S	249.29	1.43	177	60	^22^	Yes
*additives*							
DMSO	C_2_H_6_OS	78.13	1.10	19	–123	^23^	No
coumarin	C_9_H_6_O_2_	146.14	1.39	70	–57	22	No
salicylic acid	C_7_H_6_O_3_	138.12	1.48	158	–38	^24^	Yes
*o*-terphenyl	C_18_H_14_	230.31	1.24	57	–27	^25^	No
antipyrine	C_11_H_12_N_2_O	188.23	1.26	110	–22	21	No
benzamide	C_7_H_7_NO	121.14	1.29	127	–10	21	Yes
nicotinamide	C_6_H_6_N_2_O	122.12	1.40	129	–8	22	Yes
tolbutamide	C_12_H_18_N_2_O_3_S	270.35	1.24	128	4	21	Yes
isonicotinamide	C_6_H_6_N_2_O	122.12	1.37	157	11	22	Yes
chlorpropamide	C_10_H_13_ClN_2_O_3_S	276.74	1.42	130	16	21	Yes
flufenamic acid	C_14_H_10_F_3_NO_2_	281.23	1.47	135	17	21	Yes
tolfenamic acid	C_14_H_12_ClNO_2_	261.71	1.42	215	63	21	Yes
sulfathiazole	C_9_H_9_N_3_O_2_S_2_	255.31	1.52	201	66	22	Yes
griseofulvin	C_17_H_17_ClO_6_	352.77	1.37	216	88	24	No
theophylline	C_7_H_8_N_4_O_2_	180.17	1.52	272	94	21	Yes

a Values are provided for the polymorph studied here, not necessarily for the most stable one.

b Source of data for *T*_g_ with * referring to this work.

c “Yes” means that the molecule can donate hydrogen atoms to form strong intermolecular hydrogen bonds.

d Materials are listed in order of their increasing *T*_g_s.

About 50–100 μL of a stock solution of a guest molecule (4–10 mg of material dissolved in 0.2–1 mL of ethanol; some of these stock solutions were further diluted by ethanol) was added to 100 mg of host material on a watch glass. To express concentrations of mixtures in mol % and vol %, the concentrations of the solutions were calculated based on molecular weights or densities of compounds, respectively (Table 1). The material became thoroughly wet but without the formation of pools of liquid. After ethanol evaporation overnight, the powder was placed into a vial and vortexed to get homogeneous distribution of components.

Several milligrams of each mixture were sandwiched between two 0.1 mm thick cover slides and melted at a temperature of 10–20 °C above the melting point *T*_m_ of the highest melting point material in a mixture. This resulted in formation of a few μm liquid film. Then, the slide was quickly transferred into a host stage (Model FP90, Mettler-Toledo) and crystallization was performed over a wide range of temperatures between the melting point and room temperature. For some hosts, crystallization was performed in a refrigerator or a freezer. The growth rate was determined as the displacement of the growth front divided by the elapsed time using polarized light optical microscopes, an Olympus BX50 and BX53 equipped with digital cameras. The melting point for each mixture was determined by observing the melt–crystal interface displacement over time at constant temperatures. The growth rate measurements for each mixture were performed on at least three different samples. To minimize material decomposition, growth rates were measured only immediately after the sample preparation, and the material was never remelted.

Differential scanning calorimetry (DSC) was performed on a Pyris-1 differential scanning calorimeter (PerkinElmer, Wellesley, Massachusetts). Materials were heated and cooled at a rate of 10 °C/min. An indium standard was used to calibrate the instrument, and nitrogen was used as the purge gas. The data were analyzed using the PerkinElmer software to extract glass-transition temperatures, melting points, and heats of fusion.

Polymorphs were identified using polarized light optical microscopy as well as powder X-ray diffraction analysis using a Bruker D8 Discover General Area Detector Diffraction System (GADDS) equipped with a VÅNTEC-2000 2D detector and a Cu-*K*α source (λ = 1.54178 Å). The X-ray beam was monochromated with a graphite crystal and collimated with a 0.5 mm capillary collimator (MONOCAP). X-ray powder diffraction patterns were collected from powder loaded into 0.8 mm Kapton capillaries in transmission mode or from as-grown crystalline films on glass slides in reflection mode.

## Results

Carbamazepine (CMZ) forms six polymorphs but crystallization from the melt is dominated by form I.^22^ Formation of this polymorph was monitored over the whole range of temperatures between *T*_m_ and room temperature. Close to the melting point, CMZ forms needle-like crystals (Figure 1A), but at lower temperatures, it crystallizes as open and then compact spherulites (Figure 1B,C). As the temperature approaches *T*_g_, the growth rates become less homogeneous and the growth front is typically not flat (Figure 1D). Similar patterns are observed for the crystallization of CMZ in the presence of additives (Figure 1E,F). Variations of growth rates close to *T*_g_ resulting in a not smooth growth front also lead to different parts of spherulites or different spherulites and growth rate scattering observed in all kinetic curves. Growth rates of CMZ in the absence and presence of coumarin additive are shown in Figure 2. Above ∼130 °C, the growth rate is not affected by the coumarin additive, but at lower temperatures it becomes higher compared to that of CMZ without additives. For the same crystallization temperature (we used *T* = *T*_g_ + 30 = 80 °C but the same trend is observed for other temperatures), the growth rate increases almost exponentially between 0 and 2 mol % of coumarin. At higher concentrations, it becomes constant (Figure 3A). The melting point of the mixtures decreases roughly linearly as the coumarin concentration rises to ∼4 mol % with a plateau observed at coumarin concentrations >6 mol % (Figure 3B). Melting points for each of two components ideally mixing in a liquid phase but immiscible in a solid state (eutectic crystallization) can be described by eq 1.^26^ In this equation, *T*_m,host_ is the melting point of the host material without additives (191.2 °C for CMZ), Δ*H*_m,host_ is a heat of fusion of the host material without additives (25.5 kJ/mol for CMZ^21^), *x* is the molar fraction of the additive, and *R* is a universal gas constant.

1 $$\frac{1}{T_{m}}=\frac{1}{T_{m,host}}-\frac{R\ln\left(1-x\right)}{\Delta H_{m,host}}$$

**Figure 1:**
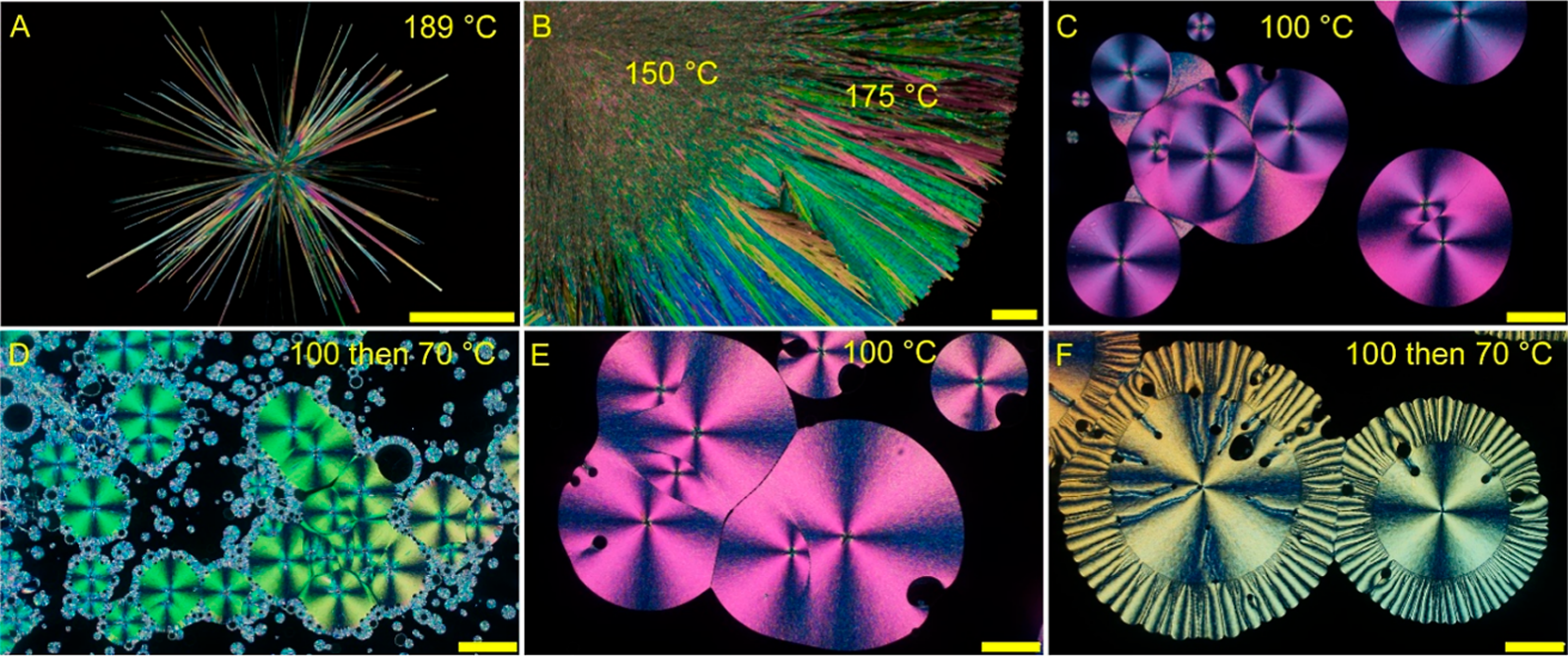
Optical micrographs of carbamazepine polymorph I growth morphologies recorded under cross polarizers. (A–D) No additives. (E,F) Additive of coumarin, 2 mol %. Crystallization temperatures are indicated in the corresponding images. Time of the growth at 70 °C for the outer parts of spherulites is 18 h for panel (D) and 5 min for panel (F). All scale bars are 0.2 mm.

**Figure 2:**
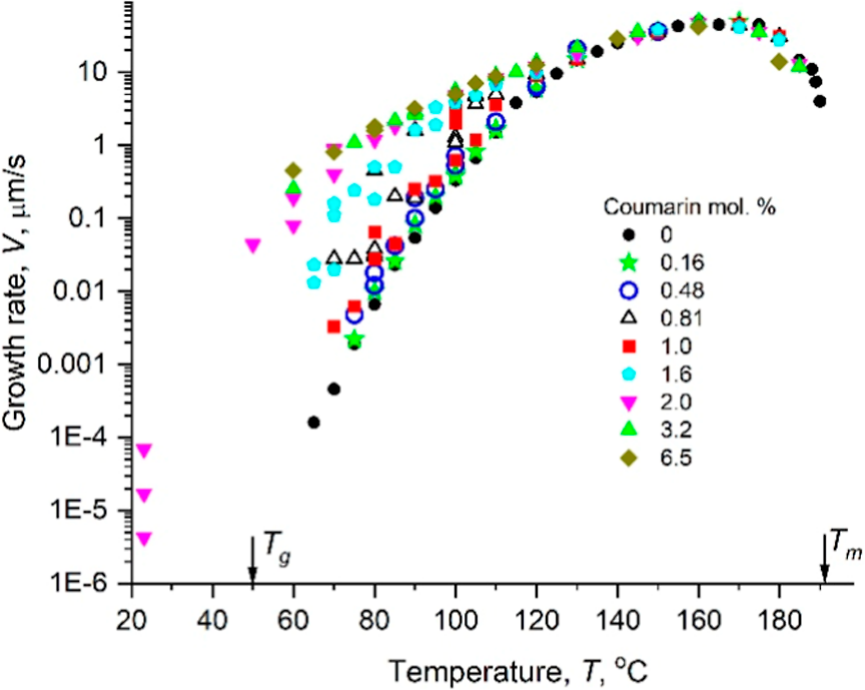
Growth rate of CMZ as a function of crystallization temperature for different concentrations of coumarin in the mixture.

**Figure 3:**
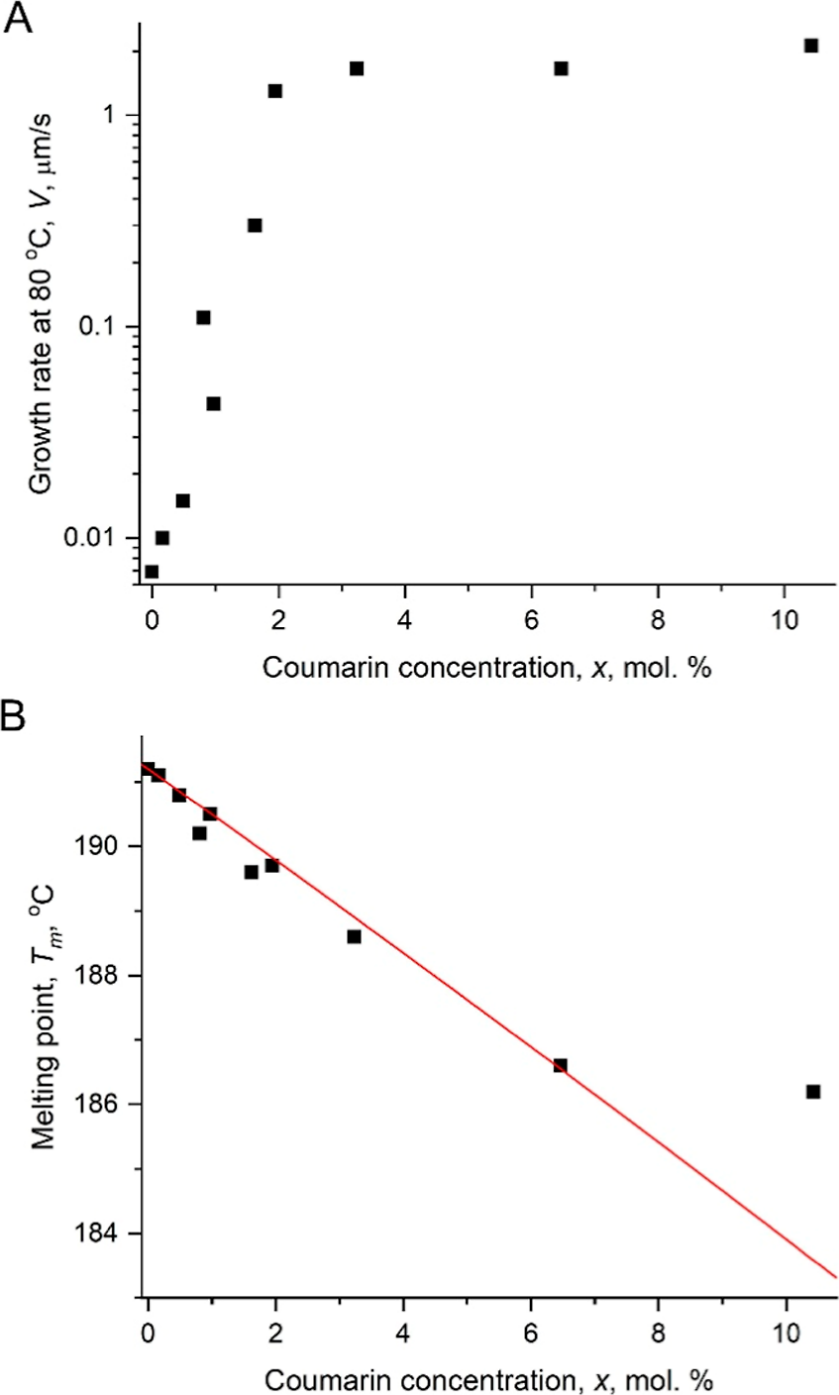
(A) Growth rate of CMZ at crystallization temperature *T* = 80 °C as a function of coumarin fraction in a mixture. (B) Melting point of CMZ crystals, *T*_m_, forming from CMZ–coumarin mixtures as a function of coumarin fraction in a mixture. Red line is calculated with eq 1.

A decent agreement between experiment and eq 1 is observed up to ∼6 mol % of coumarin with the maximum differences in *T*_m_ up to 0.4 °C (experimental error of *T*_m_ determination is 0.1–0.2 °C). At higher coumarin concentrations this agreement breaks down suggesting liquid–liquid phase separation. Even though a plateau for growth rates (Figure 3A) occurs at lower coumarin concentration *x* = 2 mol %, given experimental errors in *T*_m_(*x*) and an approximate character of eq 1 one can speculate that the growth rate stops increasing at the onset of liquid–liquid phase separation, when the melt reaches a maximum additive concentration.

Other 18 small-molecule additives tested revealed that below 130 °C some additives increased the growth rate while others did not affect it much or even slightly slowed down (Figure 4). The effect was found to be directly related to the *T*_g_ of the additive compared to the *T*_g_ of CMZ (Figure 5).

**Figure 4:**
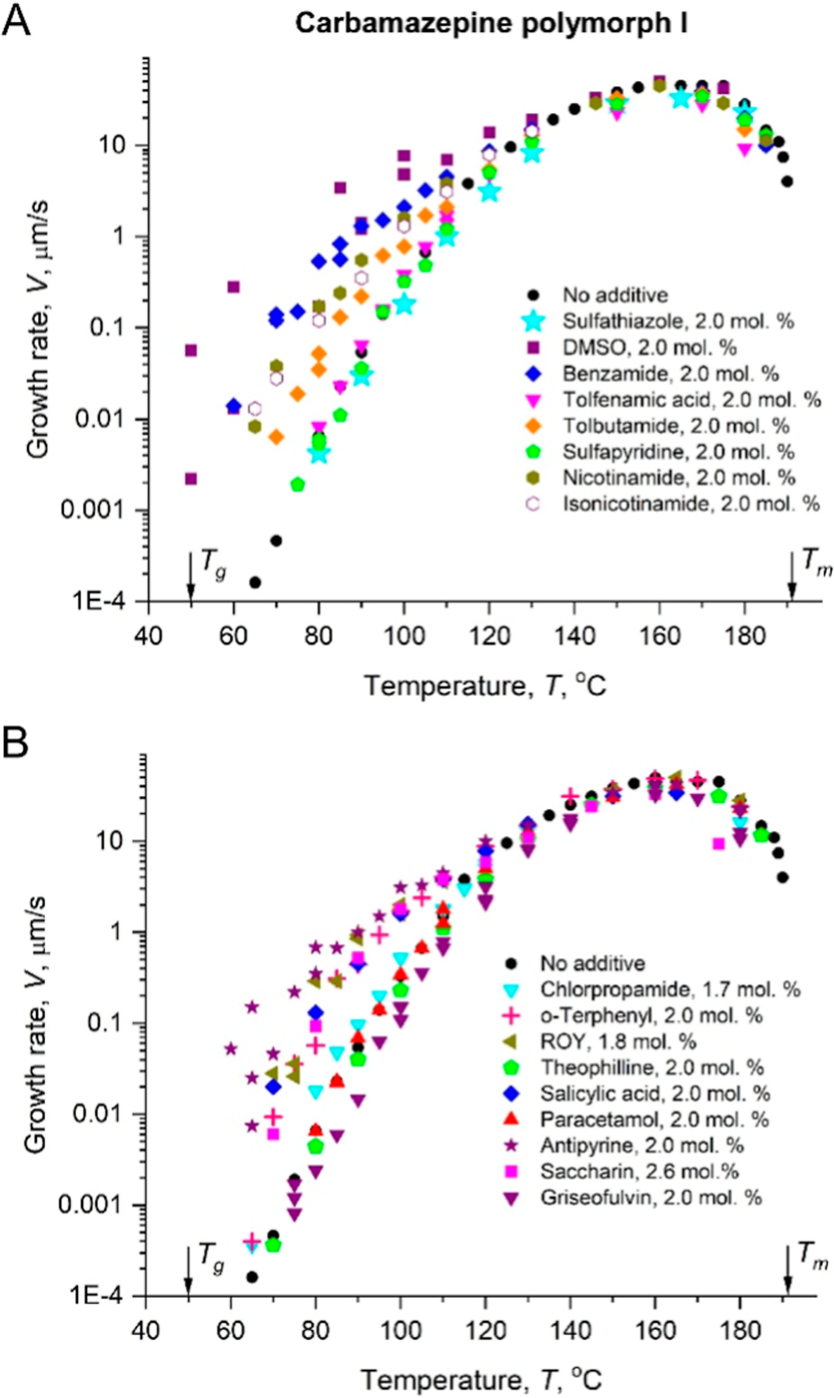
Growth rate of CMZ as a function of crystallization temperature in the presence of ∼2 mol % of additives.

**Figure 5:**
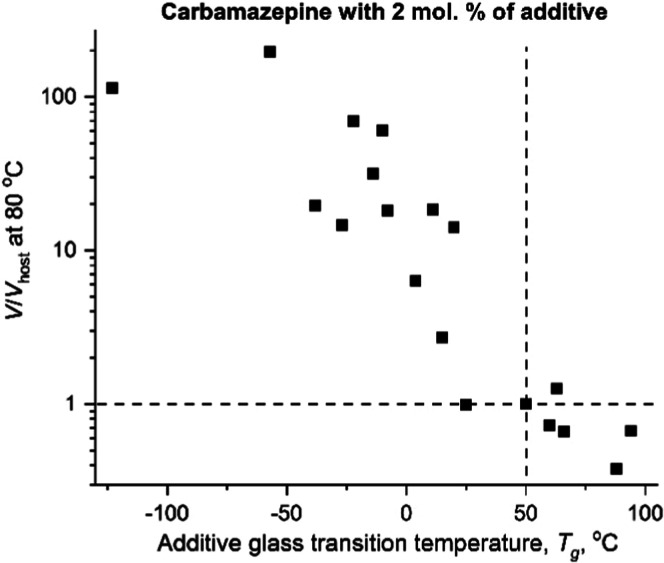
Growth rate of CMZ with ∼2 mol % of additive normalized to the growth rate of CMZ without additive, measured at 80 °C, as a function of the additive *T*_g_. The dashed lines cross at the point corresponding to CMZ without additives. See Table S1 for the tabulated raw data.

Similar, albeit less comprehensive studies were performed for 6 other materials (Figure 6). The strongest effect was observed for sulfapyridine polymorph VII^22^ (Figure 6A). The magnitude of growth rate change by the additive is smaller than for CMZ but it also can be correlated with *T*_g_ of the additive (*V*/*V*_host_ was calculated for *T* = *T*_g_ + 30 = 90 °C for easier comparison with CMZ) (Figure 7). Crystallization of paracetamol polymorph I was complicated by the so-called glass-to-crystal (GC) growth mode.^27^ This mode is characterized by substantial increase in the growth rate at temperatures around *T*_g_ and, at slightly higher growth temperatures, by formation of loose fibers growing much faster than the majority of the crystallites.^25,28,29^ Growth rates of paracetamol were substantially decreased by griseofulvin (Figure 6B), whose *T*_g_ is 63 °C higher than *T*_g_ of paracetamol in agreement with the trend observed for CMZ and sulfapyridine. Other additives did not show any effect at 2 mol %, but coumarin showed some growth rate increase at 4 mol %. No changes in the growth rate were detected for deltamethrin polymorph I (Figure 6C), α-cypermethrin (Figure 6D), chlorfenapyr polymorph I^18^ (Figure 6E), and ROY polymorph YN^19^ (Figure 6F) at *x* ≈ 2 mol %. At higher concentrations, a growth rate increase became evident though (Figure 6C).

**Figure 6:**
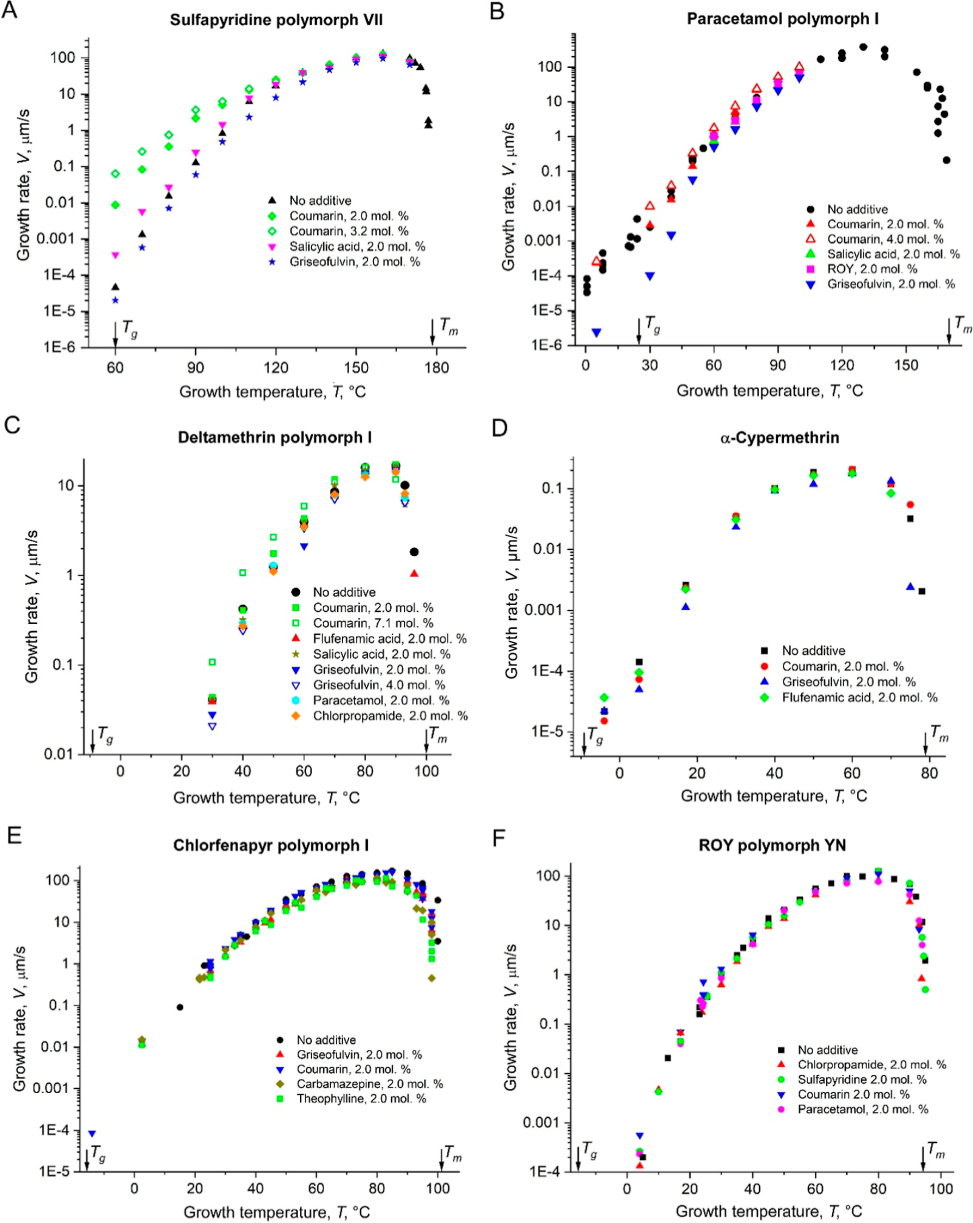
Growth rate of sulfapyridine (A), paracetamol (B), deltamethrin (C), α-cypermethrin (D), chlorfenapyr (E), and ROY (F) as a function of crystallization temperature in the presence of additives.

**Figure 7:**
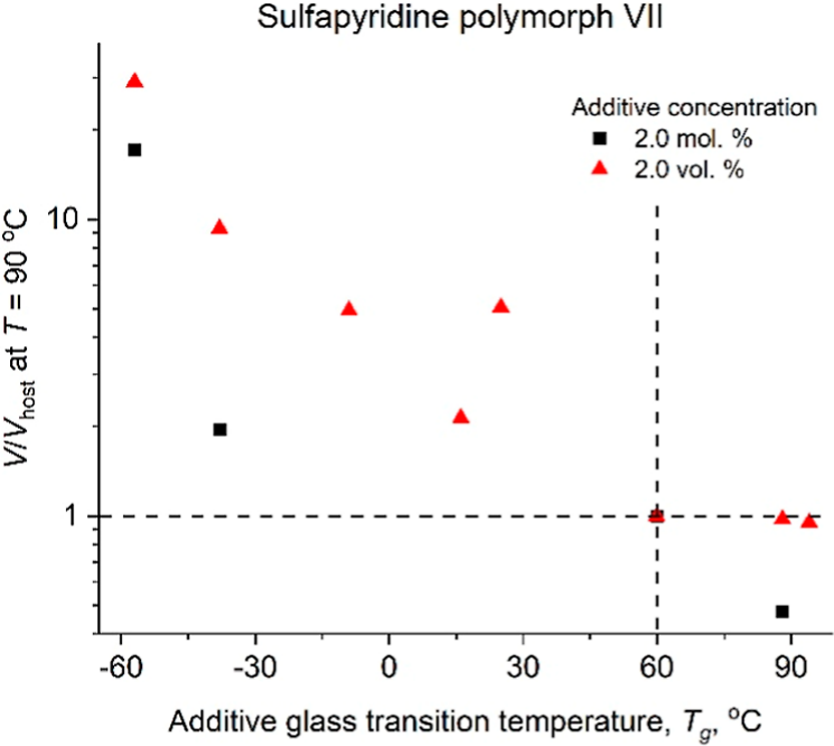
Growth rate of sulfapyridine polymorph VII with 2 mol % and 2 vol % of additives normalized to the growth rate of sulfapyridine without additive, measured at 90 °C, as a function of the additive *T*_g_. The dashed lines cross at the point corresponding to sulfapyridine without additives. See Table S2 for the tabulated raw data.

Since host and additive molecules can have different sizes, it would be more correct to compare the effect of different additives by measuring kinetic curves at constant volume fraction of the additive, not constant molar fraction. This caveat was understood when most of the data were already collected. For that reason, we compared results for constant molar fraction (2 mol %) with the results for constant volume fraction (2 vol %) only for one host and very few additives. The data for 2 vol % show better correlation between *V*/*V*_host_ and *T*_g,additive_-*T*_g,host_ supporting our idea (Figure 7).

## Discussion

The key findings are summarized as follows. Small concentrations (*x* ≈ 2 mol %) of small molecular additives can substantially (up to a few orders of magnitude) change the growth rate of molecular crystals. The magnitude of the *V*/*V*_host_ ratio increases as the additive concentration increases but only as long as the host and the additive are completely miscible in the melt. Growth rates are affected only within several tens of degrees above the host’s *T*_g_. The magnitude of the effect as well as growth rate dispersion increase as temperature decreases and approaches *T*_g_. The additives with *T*_g,additive_ > *T*_g,host_ lead to growth rate decrease *V*/*V*_host_ < 1, while the additives with *T*_g,additive_ < *T*_g,host_ lead to growth rate increase *V*/*V*_host_ > 1. For the same additive concentration and absolute |*T*_g,additive_–*T*_g,host_| difference, the effect is much stronger for the additives with *T*_g,additive_ < *T*_g,host_ compared to the additives with *T*_g,additive_ > *T*_g,host_. Randomly distributed additive molecules of similar size at *x* ≈ 2 mol % are separated by about *x* ^–1/3^–1 or 2–3 host molecules assuming not much direct additive–additive interaction.

All molecules examined herein have hydrogen bond acceptors, but only several of them have strong hydrogen bond donors (Table 1) meaning that only these molecules can form hydrogen bonds in the melt. Out of seven host molecules studied, only three can form intermolecular hydrogen bonds in the melt and only these molecules reveal substantial growth rate changes in the presence of additives.

The growth rate in melt crystallization can be described by eq 2,^30^ where *V*_0_ is a temperature-independent term and Δ*G*_V_ is a deviation from thermodynamic equilibrium, which can be approximately calculated as Δ*G*_V_ = Δ*H*_m_(*T*_m_–*T*)/*T*_m_. *D*(*T*) is a term related to the molecular mobility in the liquid. *D*(*T*) is proportional to the diffusion coefficient for transport across the melt–crystal interface or is inversely proportional to the liquid viscosity. For strong liquids, viscosity η fits well to the Arrhenius equation but more common fragile liquids are better described by the Vogel–Tammann–Fulcher (VTF) equation.^31,32^

2 $$V=V_{0}D\left(T\right)\left[1-\exp\left(-\frac{\Delta G_{V}}{RT}\right)\right]$$

Growth rate changes discussed in this paper become significant far below *T*_m_ (Figures 2, 4, and 7), where the term in square brackets in eq 2 is close to 1. Therefore, the effect of additives on the growth rate is kinetic by nature and is related to molecular mobility *D*(*T*).

We speculate that the additives disrupt bonding between host molecules, creating areas with either higher or lower mobility. Since the average distance between additive molecules is only two to three host molecules, disruption of the closest neighbors efficiently changes mobility for the majority of host molecules. The interactions between the molecules are easier to disrupt if they correspond to more localized hydrogen bonds compared to dispersive interactions. This explains why a growth rate change occurs in host molecules capable of forming intermolecular hydrogen bonds.

At any given temperature, mobility is lower if the molecules in the liquid are more strongly bound to one another, and their reorganization requires higher energy. If *T*_g,additive_ < *T*_g,host_, then the additive will move more actively in the melt, make surrounding molecules more mobile too and increase the growth rate. In the opposite situation of *T*_g,additive_ > *T*_g,host_, the growth rate should decrease. A more mobile molecule can more easily increase the mobility of the surrounding host molecules, while a less mobile molecule located among more mobile ones should have a weaker effect on the surrounding molecules. This logic is consistent with the experimental observations. The effect of additives on the growth rate becomes stronger as temperature approaches *T*_g_, likely because a liquid is spatially more heterogeneous at lower temperatures,^33,34^ providing more pathways for higher/slower mobility.

## Conclusions

Significant change in growth rates of molecular crystals in the presence of small molecular additives was observed in crystallization from the melt close to *T*_g_. The magnitude of growth rate change is correlated with the difference between *T*_g_ of the host and the additive, and the effect is stronger for the host materials forming intermolecular hydrogen bonds. This phenomenon does not have a straightforward explanation but it should be related to the supercooled liquid dynamics and disruption of the liquid structure by the additive with a different mobility. Further understanding can be potentially gained by the simulations of the liquid dynamics in the presence of additives.

## Supplementary Material



## Notes

Molecular structures of hosts/additives and tabulated raw data used for plotting Figures 5 and 7 (PDF)
