# Supplementary material for: Effect of Small Molecular Additives on Growth Rates of Molecular Crystals from the Melt near Glass-Transition Temperature
Source: Cryst Growth Des. 2025 Dec 15;26(1):494–500. doi: 10.1021/acs.cgd.5c01407 (PMC12784327; doi:10.1021/acs.cgd.5c01407)
Supplement: Supplementary file 1 [file cg5c01407_si_001.pdf]

Supporting Information

Effect of small molecular additives on growth rates of molecular crystals from the melt near glass transition temperature

Alexander G. Shtukenberg, Hengyu Zhou, Eli Finkelstein, Aminata Dioume

Department of Chemistry and Molecular Design Institute, New York University, New York, NY 10003, USA

Table of content

Figure S1. Molecular structures of hosts/additives used in this research.

Table S1. Tabulated raw data plotted in Figure 5.

Table S2. Tabulated raw data plotted in Figure 7.

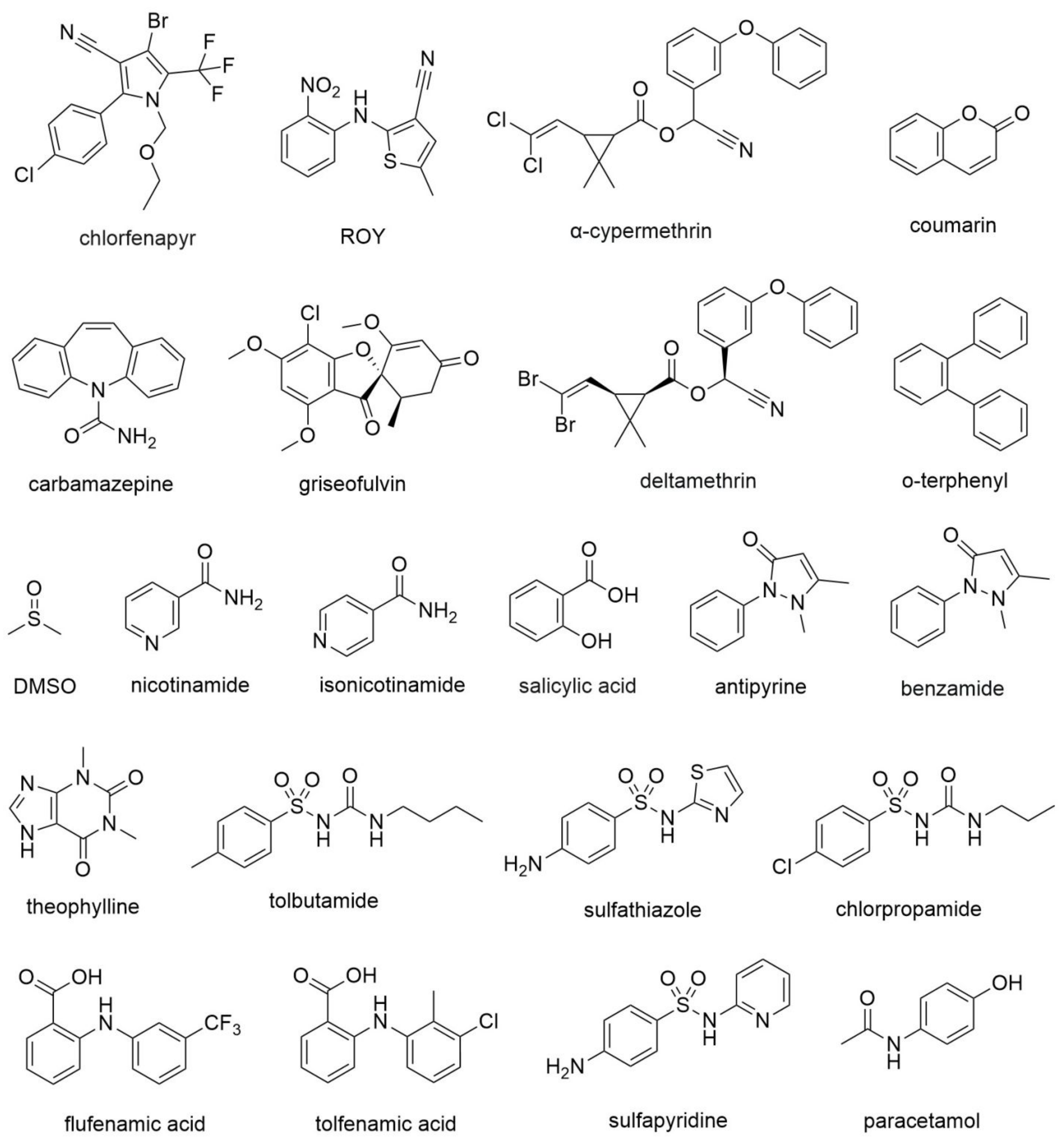

Figure S1. Molecular structures of hosts/additives used in this research (Table 1).

Table S1. Tabulated raw data plotted in Figure 5. All additives have 2 mol. % concentrations and all measurements were performed at 80 °C. Growth rate of carbamazepine host is 0.0066 μm/s.

Additive	Growth rate, V, μm/s	V/V_{host}
Chlorpropamide	0.018	2.72
Isonicotinamide	0.121	18.3
Nicotinamide	0.12	18.2
Saccharin	0.093	14.1
Coumarin	1.3	197
ROY	0.21	31.8
Sulfapyridine	0.0048	0.727
Sulfathiazole	0.0044	0.667
DMSO	0.752	114
Theophylline	0.00441	0.668
Salicylic Acid	0.13	19.7
Tolfenamic Acid	0.0083	1.26
Antipyrine	0.46	69.7
Benzamide	0.401	60.7
Tolbutamide	0.042	6.36
Paracetamol	0.0065	0.985
o-Terphenyl	0.096	14.5
Griseofulvin	0.0025	0.379

Table S2. Tabulated raw data plotted in Figure 7. All measurements were performed at 90 °C. Growth rate of sulfapyridine VII host is 0.126 μm/s.

Additive	Additive concentration 2.0 vol. %		Additive concentration 2.0 mol. %	
	Growth rate, V, mm/s	V/V_{host}	Growth rate, V, mm/s	V/V_{host}
griseofulvin	0.123	0.976	0.06	0.476
coumarin	3.66	29.0	2.156	17.1
salicilyc acid	1.18	9.33	0.247	1.96
α-cypermethrin	0.623	4.94	No data	No data
chlorpropamide	0.27	2.14	No data	No data
paracetamol	0.638	5.06	No data	No data
theophylline	0.12	0.952	No data	No data
